# ADC Histogram Features of Breast Cancer Brain Metastases as Candidate Imaging Biomarkers of Primary Tumor ER, PR, Ki-67, and Luminal Status

**DOI:** 10.3390/diagnostics16081154

**Published:** 2026-04-13

**Authors:** Diba Saygılı Öz, Burcu Savran, Nazan Çiledağ, Özkan Ünal, Berna Karabulut

**Affiliations:** 1 Radiology Department, Dr Abdurrahman Yurtaslan Ankara Oncology Training and Research Hospital, Demetevler, 354/1. Sk., Yenimahalle, Ankara 06200, Türkiye; 2 Pathology Department, Dr Abdurrahman Yurtaslan Ankara Oncology Health Application and Research Hospital, Demetevler, 354/1. Sk., Yenimahalle, Ankara 06200, Türkiye

**Keywords:** apparent diffusion coefficient, brain metastases, breast cancer, diffusion-weighted imaging, Ki-67, logistic regression

## Abstract

**Background**: Breast cancer brain metastases (BCBMs) are clinically challenging, and treatment decisions are influenced by tumor biology. Because receptor profiles may differ between primary breast tumors and brain metastases and brain biopsy may be impractical, non-invasive imaging biomarkers may provide useful biologic correlates. We evaluated whether diffusion-weighted imaging (DWI)-derived apparent diffusion coefficient (ADC) histogram metrics from BCBM were associated with primary tumor estrogen receptor (ER), progesterone receptor (PR), and human epidermal growth factor receptor 2 (HER2) status; the Ki-67 proliferation index; and luminal status. **Methods**: This retrospective exploratory single-center study included 72 adults with BCBM who underwent standardized 1.5T brain magnetic resonance imaging. The largest lesion in each patient was segmented on ADC maps in FireVoxel. ADC histogram features, including percentiles, were extracted. Using primary tumor biomarker status as the reference, candidate metrics were screened by univariable logistic regression. Parsimonious multivariable models included age, log-transformed lesion volume, and a single selected ADC percentile scaled by ×10. Discriminatory performance was assessed using area under the receiver operating characteristic curve (AUC); thresholds were derived with the Youden index. No external validation was performed. **Results**: Low-percentile ADC metrics were associated with ER positivity, PR positivity, and luminal disease, whereas no meaningful ADC histogram discrimination was observed for HER2. In multivariable models, ADC10×10 predicted ER positivity (odds ratio [OR] 0.441; AUC 0.847) and PR positivity (OR 0.478; AUC 0.819). Ki-67 positivity was best predicted by ADC75×10 (OR 3.095; AUC 0.905), although this finding should be interpreted cautiously. Luminal status (non-luminal vs. luminal) was predicted by ADC10×10 (OR 2.251; AUC 0.832). **Conclusions**: ADC histogram analysis from DWI in BCBM showed exploratory associations with primary tumor hormone receptor status and luminal subtype, but not HER2. These findings support ADC histogram features as candidate imaging biomarkers, but the Ki-67 result and all model performance estimates require cautious interpretation and independent external validation in multicenter cohorts.

## 1. Introduction

Breast cancer brain metastases (BCBMs) represent a clinically challenging stage of disease in which treatment selection is influenced by tumor biology, particularly estrogen receptor (ER), progesterone receptor (PR), and human epidermal growth factor receptor 2 (HER2) status. Receptor discordance between the primary breast tumor and brain metastases has been reported in BCBM cohorts, with substantial conversion rates and frequent hormone receptor loss, highlighting the clinical importance of biologic reassessment when feasible [1,2,3]. At the same time, tissue sampling of brain metastases is not always practical or safe, which has increased interest in non-invasive imaging biomarkers. In this setting, however, it is important to distinguish between two related but distinct questions: whether imaging can estimate the biologic status of the metastasis itself, and whether imaging features of brain metastases are associated with the biomarker status of the primary breast tumor.

Diffusion-weighted imaging (DWI)-derived apparent diffusion coefficient (ADC) mapping is widely available in routine magnetic resonance imaging (MRI) protocols and provides information related to tissue microstructure and water diffusivity. In primary breast cancer, ADC-based metrics, including whole-lesion histogram features, have shown associations with receptor status, Ki-67, and molecular subtype in several studies and reviews [4,5]. However, these relationships are not deterministic. Prior syntheses and multicenter analyses have also shown substantial overlap in ADC values across breast cancer subtypes, limiting discrimination at the individual level and suggesting that conventional diffusion metrics should be interpreted cautiously as biologic surrogates [6,7].

Evidence specific to BCBM remains limited. To our knowledge, only one dedicated ADC histogram study has directly evaluated breast cancer brain metastases. In that study, lower ADC percentiles were associated with ER/PR-positive disease, whereas no significant association was found for HER2. Importantly, the results varied according to whether the analysis included only the largest metastasis or all measurable metastases per patient [8]. This distinction is methodologically relevant in metastatic disease, where intrapatient heterogeneity may be substantial and lesion selection strategy may influence the observed associations. More broadly, multiparametric MRI radiomics studies in BCBM have reported higher performance for receptor or subtype classification than ADC histogram features alone, suggesting that conventional ADC metrics may capture only part of the biologic signal, particularly for HER2-related characterization [1,2,3].

Direct BCBM evidence for Ki-67 is even more limited. The dedicated BCBM ADC-histogram study did not evaluate Ki-67, and prior diffusion-based observations on proliferation have largely come from primary breast cancer or mixed brain metastasis cohorts rather than breast-only brain metastases [4,5,8]. Accordingly, any assessment of Ki-67-related diffusion patterns in BCBM should be considered preliminary. Similar caution applies to luminal characterization, especially when luminality is operationalized in a simplified manner using hormone receptor positivity rather than contemporary intrinsic subtype classification.

Against this background, the aim of this retrospective exploratory study was to assess whether ADC histogram features derived from the largest breast cancer brain metastasis are associated with primary tumor ER, PR, HER2, and Ki-67 status, as well as with a simplified luminal classification defined as ER and/or PR positivity versus ER/PR negativity. Framed in this way, the study was designed to evaluate whether routine DWI-derived ADC histogram features may serve as candidate imaging correlates of primary-tumor biology in BCBM while acknowledging the limited BCBM-specific literature and the need for cautious interpretation and further validation.

## 2. Materials and Methods

### 2.1. Study Design and Ethics

This retrospective study was approved by the Institutional Review Board of a training and research hospital (Approval No.: 2021-04/1121 and 2022-01/1121). Written informed consent had been obtained from all participants at the time of clinical care in accordance with institutional procedures and the Declaration of Helsinki.

### 2.2. Patient Selection

The study was conducted between April 2021 and February 2022 at the Department of Radiology, Dr. Abdurrahman Yurtaslan Ankara Oncology Training and Research Hospital. For patient selection, cranial MRI examinations performed between January 2019 and March 2021 were screened retrospectively in the radiology archive.

**Figure 1:**
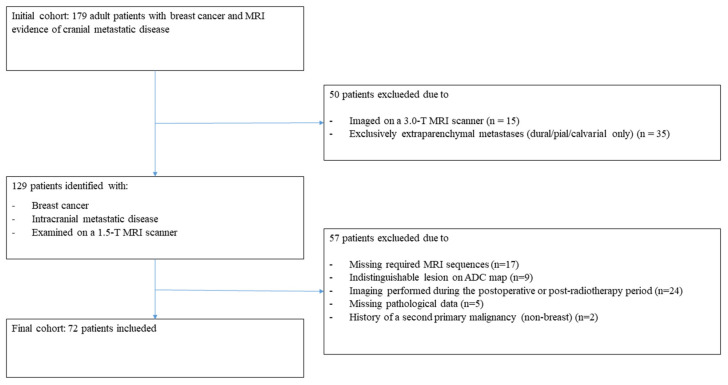
Study diagram for the inclusion of patients in the analysis.

Cranial MRI examinations were reviewed from the institutional radiology archive. Adult patients (≥18 years) with a diagnosis of breast cancer and radiologically confirmed intracranial metastatic disease were screened. The exclusion criteria for these patients are summarized in Figure 1. Because of the retrospective design, no a priori sample size calculation was performed; all consecutive eligible patients examined with the specified contrast-enhanced brain MRI protocol during the study period were considered. Patients were excluded if MRI was incomplete or not suitable for diffusion/ADC analysis, if pathologic biomarker information was unavailable, or if imaging–pathology matching was uncertain (Figure 1).

The final analytic dataset comprised 72 patients used for statistical analyses. Each observation corresponded to an intracranial metastatic lesion selected for post-processing according to the lesion selection strategy described below.

### 2.3. MRI Acquisition

All patients were imaged using a 1.5 Tesla MRI scanner (Signa Explorer; GE Healthcare, Milwaukee, WI, USA) with a 16-channel head coil. To reduce measurement variability, examinations were acquired using a standardized institutional brain metastasis protocol (Supplementary Table S1), including diffusion-weighted imaging and post-contrast sequences. Diffusion-weighted imaging was performed using an axial spin-echo echo-planar imaging (SE-EPI) sequence with b values of 0 and 1000 s/mm^2^ in three diffusion-encoding directions. The repetition time/echo time (TR/TE) was 7702/93.20 ms, slice thickness/intersection gap was 4/1 mm, field of view was 22 × 22 cm, and matrix size was 160 × 160. ADC maps were generated automatically from the b = 0 and b = 1000 s/mm^2^ diffusion-weighted images using the scanner’s standard reconstruction workflow. Examinations performed on 3.0-T systems were excluded to further reduce acquisition-related variability.

### 2.4. Lesion Identification and Selection

Metastatic lesions were identified on conventional MRI sequences and post-contrast images. When multiple intracranial metastases were present, we selected a single lesion per patient as a pragmatic strategy to avoid within-patient clustering and maintain analytical independence; however, we acknowledge that the largest lesion may not fully represent intrapatient metastatic heterogeneity. For ADC-based analysis, lesions were classified as non-evaluable and excluded when reliable manual delineation on DWI/ADC maps was not possible because of indistinct lesion margins, very small lesion size, severe susceptibility/distortion artifact, or poor lesion-to-background contrast. This approach is consistent with prior DWI/ADC literature and consensus recommendations emphasizing sufficient lesion conspicuity, adequate lesion size, and avoidance of major artifacts for meaningful quantitative ADC analysis [9,10,11,12].

**Figure 2:**
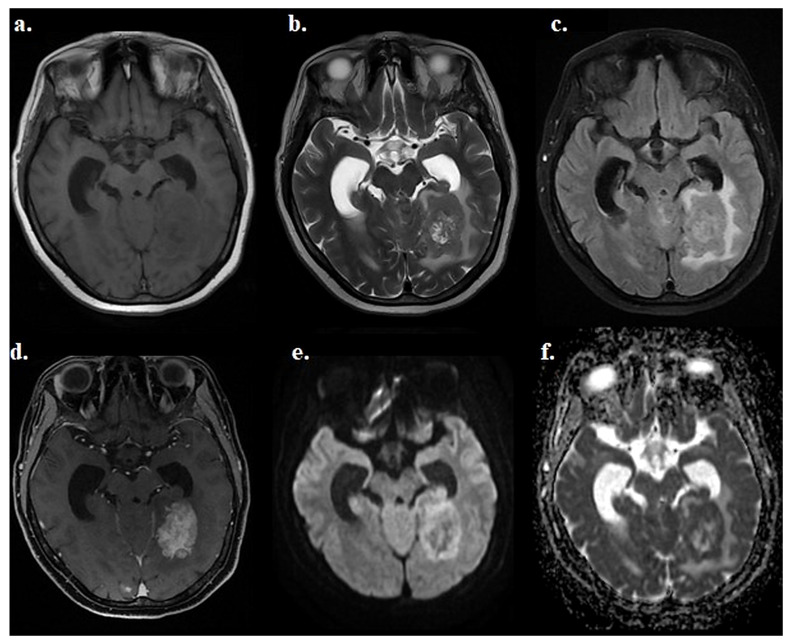
Representative multiparametric MRI appearance of a breast cancer brain metastasis. Example of a representative intraparenchymal metastatic lesion shown across routine clinical MRI sequences to illustrate lesion conspicuity and intralesional heterogeneity before post-processing. The lesion is shown on T1-weighted imaging (**a**), T2-weighted imaging (**b**), FLAIR imaging (**c**), post-contrast T1-weighted imaging (**d**), DWI (**e**), and the ADC map (**f**). The segmentation workflow and histogram extraction procedure are presented separately in Figure 3.

Figure 2 illustrates the multiparametric MRI appearance and intralesional heterogeneity of a representative metastatic lesion as assessed across routine clinical sequences.

**Figure 3:**
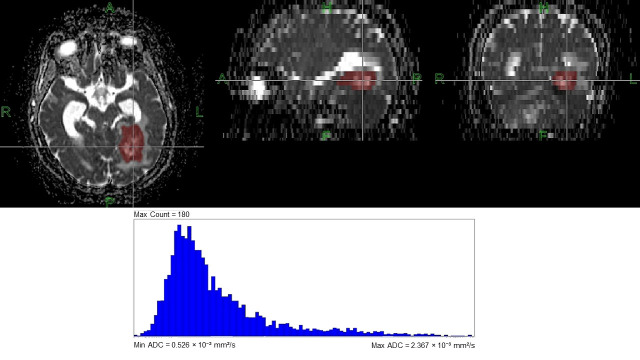
ROI drawings and ADC histogram analysis of the lesion on ADC map. ROI: region of interest; ADC: apparent diffusion coefficient. A, anterior; P, posterior; R, right; L, left; H, head; F, foot.

### 2.5. Post-Processing and ADC Histogram Analysis

ADC maps were exported for post-processing using FireVoxel software (build 364A). Regions of interest (ROIs) were manually delineated on ADC maps across contiguous slices to encompass the entire visible intraparenchymal metastatic lesion. Direct segmentation on ADC maps was chosen to avoid registration-induced error and to ensure that histogram features were extracted from the same diffusion-defined image space in which ADC was measured. A whole-lesion approach was deliberately used to preserve intralesional heterogeneity for histogram analysis, consistent with prior BCBM ADC histogram work [8]. Accordingly, cystic, necrotic, and hemorrhagic intralesional components were not intentionally excluded when present; however, obvious non-lesional CSF spaces and major contouring errors at the lesion margins were avoided during VOI construction. Volumes of interest (VOIs) were created from slice-wise ROIs and visually checked in multiple planes to ensure adequate lesion coverage. Initial segmentation and measurements were performed by a recently board-certified radiologist. For interreader reproducibility analysis, a board-certified radiologist with 13 years of experience independently repeated the segmentation and measurements in all 72 cases. The second radiologist was blinded to the first radiologist’s measurements, and both radiologists were blinded to the primary tumor histopathologic biomarker results. Once delineation was complete, histogram analysis was performed (Figure 3).

Histogram analysis was then performed on the lesion volume. The following ADC histogram features were extracted: volume, ADCmin, ADC1, ADC5, ADC10, ADC25, ADC50, ADC75, ADC90, ADC95, ADC99, ADCmax, skewness, kurtosis, and entropy. Skewness and kurtosis were obtained automatically by FireVoxel from the voxelwise ADC distribution within the VOI and correspond to the third and fourth standardized central moments, respectively (kurtosis reported as excess kurtosis). Entropy was computed by FireVoxel as Shannon entropy of the normalized ROI histogram probabilities.

To improve interpretability in regression models, selected ADC predictors were scaled by a factor of 10 (e.g., ADC10×10 and ADC75×10) so that odds ratios (ORs) correspond to a 0.1-unit change in the original ADC scale.

### 2.6. Pathological Evaluation

**Figure 4:**
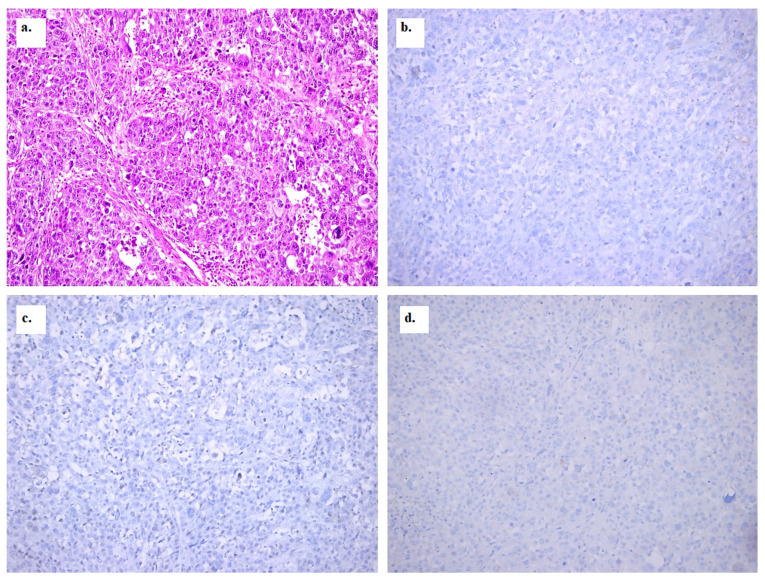
Representative histopathologic and immunohistochemical images of breast cancer brain metastasis. Representative images from the primary breast tumor used as the reference for biomarker assessment. Hematoxylin and eosin staining is shown in panel (**a**), estrogen receptor (ER) immunohistochemistry in panel (**b**), progesterone receptor (PR) immunohistochemistry in panel (**c**), and human epidermal growth factor receptor 2 (HER2) immunohistochemistry in panel (**d**). Original magnification, ×200.

Pathology results were retrieved from institutional records for the primary breast tumor. Biomarkers included estrogen receptor (ER), progesterone receptor (PR), human epidermal growth factor receptor 2 (HER2), and Ki-67. ER and PR were considered positive when immunoreactivity exceeded 1%. HER2 positivity was assessed according to the 2018 American Society of Clinical Oncology/College of American Pathologists (ASCO/CAP) guidelines using immunohistochemical and in situ hybridization techniques. Ki-67 was dichotomized using a 20% threshold (≥20% considered positive). Luminality was dichotomized as luminal (hormone receptor-positive: ER- and/or PR-positive) versus non-luminal (hormone receptor-negative: ER- and PR-negative). Representative images are given in Figure 4 for a triple-negative tumor with high-grade cytologic features (hematoxylin–eosin staining) (Figure 4a). ER and PR negativity are shown as an absence of nuclear staining (Figure 4b,c). HER2 negativity is shown as a lack of any membranous expression (Figure 4d).

### 2.7. Statistical Analysis

All statistical analyses were performed using jamovi (The jamovi project; Version 2.7) with relevant modules (ClinicoPath, MedDecide) and the embedded R environment [13,14]. Continuous variables are summarized as median (interquartile range, IQR) because normality assumptions were not met for most continuous variables (Shapiro–Wilk test, *p* < 0.05). Categorical variables are summarized as counts and percentages.

For group comparisons of continuous imaging metrics across dichotomous groups (ER, PR, HER2, and Ki-67 status, each coded as positive [P] vs. negative [N], and luminality coded as luminal [hormone receptor-positive: ER- and/or PR-positive] vs. non-luminal [hormone receptor-negative: ER- and PR-negative]), the Mann–Whitney U test was used.

To model biomarker status, binomial logistic regression was performed. Univariable models were first fitted for each ADC histogram metric to explore discriminatory potential. Because ADC percentiles represent overlapping information and may be highly correlated, multiple ADC percentiles were not entered simultaneously into the same multivariable model in order to avoid multicollinearity. Instead, for each endpoint, the final multivariable model was constructed in a parsimonious manner including age, log-transformed lesion volume (log_volume), and a single selected ADC percentile metric. Age was included as a basic patient-level covariate, whereas log-transformed lesion volume was included as an imaging covariate because lesion size may influence ADC histogram distributions through partial volume effects and intralesional heterogeneity. Log transformation was used to reduce the influence of the right-skewed lesion volume distribution. To improve numerical interpretability, selected ADC predictors were scaled (e.g., ADC10x10 = ADC10 × 10; ADC75x10 = ADC75 × 10) so that odds ratios represent the change per 0.1-unit increase in the original ADC scale.

This analysis was designed as an exploratory imaging biomarker study. Because multiple ADC histogram metrics were screened across several biological endpoints, the results are potentially susceptible to type I error and feature selection bias. No formal multiplicity correction was applied, as the primary aim was hypothesis generation rather than confirmatory inference. No resampling, class weighting, or precision–recall-based optimization was performed. Given the exploratory design and limited sample size, particularly for the Ki-67-negative subgroup, class imbalance was addressed primarily through cautious interpretation of discrimination metrics rather than algorithmic correction.

Model discrimination was assessed using receiver operating characteristic (ROC) analysis and the area under the ROC curve (AUC). For the multivariable models, predicted probabilities were exported and evaluated with ROC analysis. Optimal probability thresholds were identified using the Youden index (maximizing sensitivity + specificity − 1), and sensitivity, specificity, accuracy, and balanced accuracy were reported at the optimized cut-off. A two-sided *p* value < 0.05 was considered statistically significant. Because the multivariable models and probability thresholds were derived and evaluated within the same cohort, the resulting AUCs and Youden-optimized cut-offs should be regarded as internally derived performance estimates.

To assess measurement reproducibility, a second radiologist independently repeated lesion segmentation and ADC histogram extraction. Interobserver agreement was evaluated for the primary imaging predictors retained in the final multivariable models (ADC10 and ADC75) and for lesion volume, rather than for the full set of screened histogram metrics, because the reproducibility analysis was intended to focus on the features ultimately carried forward into model-based interpretation. ICC values were interpreted using commonly applied thresholds (<0.50 poor, 0.50–0.75 moderate, 0.75–0.90 good, and >0.90 excellent agreement).

## 3. Results

### 3.1. Cohort Characteristics

A total of 72 observations were analyzed. Median age was 54.0 years (IQR 15.25; range 33–77). Median lesion volume was 1.102 (IQR 6.599; range 0.046–114.09). Baseline characteristics of the cohort and lesions are summarized in Table 1.

### 3.2. Group Comparisons

ER group comparisons: In nonparametric comparisons (Mann–Whitney U; ER-negative [N, *n* = 32] vs. ER-positive [P, *n* = 40]), lesion volume differed significantly between groups (*p* = 0.021; rank-biserial r = −0.318), with smaller volumes in ER-positive lesions (median 0.754 vs. 4.319). Across diffusion histogram metrics, all ADC percentile measures and ADCmax were significantly lower in ER-positive lesions (ADCmin *p* = 0.024; ADC1–ADC99 all *p* < 0.001; ADCmax *p* = 0.001; rank-biserial r ≈ −0.311 to −0.687), consistent with descriptives showing uniformly lower mean/median ADC values in the ER-positive group. Among shape descriptors, skewness was higher in ER-positive lesions (*p* = 0.032; r = 0.297), whereas kurtosis (*p* = 0.650) and entropy (*p* = 0.242) did not differ significantly between ER groups.

PR group comparisons: In nonparametric comparisons (Mann–Whitney U; PR-negative [N, *n* = 33] vs. PR-positive [P, *n* = 39]), lesion volume differed significantly between groups (*p* = 0.019; rank-biserial r = −0.323), with smaller volumes in PR-positive lesions (median 0.753 vs. 4.278). Across diffusion histogram metrics, all ADC percentile measures and ADCmax were significantly lower in PR-positive lesions (ADCmin *p* = 0.021; ADC1–ADCmax all *p* < 0.001; rank-biserial r ≈ −0.317 to −0.660), consistent with descriptives showing uniformly lower mean/median ADC values in the PR-positive group. Among shape descriptors, skewness was modestly higher in PR-positive lesions (*p* = 0.047; r = 0.274), whereas kurtosis (*p* = 0.422) and entropy (*p* = 0.114) did not differ significantly between PR groups.

HER2 group comparisons: In nonparametric comparisons (Mann–Whitney U; HER2-negative [N, *n* = 40] vs. HER2-positive [P, *n* = 32]), lesion volume showed only a borderline difference between groups (*p* = 0.066; rank-biserial r = 0.255), with numerically larger volumes in HER2-positive lesions (median, 3.003 vs. 0.874). No ADC percentile metric differed significantly by HER2 status (ADCmin, *p* = 0.964; ADC1-ADCmax, *p* = 0.318–0.515). Effect sizes were also uniformly small across the evaluated ADC metrics (rank-biserial r range, −0.007 to 0.139) (Supplementary Table S2), and histogram shape metrics (skewness, kurtosis, and entropy) likewise showed no significant between-group differences (*p* = 0.579, 0.812, and 0.757, respectively). Overall, these findings indicate no meaningful or consistent ADC histogram signal for distinguishing HER2-positive from HER2-negative lesions in this cohort.

Ki-67 group comparisons: In nonparametric group comparisons (Mann–Whitney U; Ki-67-negative [N, *n* = 9] vs. Ki-67-positive [P, *n* = 63]), lesion volume did not differ significantly between groups (*p* = 0.121). In contrast, all ADC percentile metrics and ADCmax were significantly higher in Ki-67-positive lesions (ADCmin *p* = 0.045; ADC1 *p* = 0.003; ADC5–ADCmax all *p* < 0.001), consistent with the descriptive statistics showing higher mean/median ADC values across the distribution in the Ki-67-positive group. Histogram shape metrics (skewness, kurtosis, entropy) did not differ between Ki-67 groups (*p* = 0.658, 0.695, and 0.563, respectively).

Luminality group comparisons: In nonparametric comparisons (Mann–Whitney U; luminal [L, *n* = 43] vs. non-luminal [N, *n* = 29]), lesion volume showed only a borderline difference between groups (*p* = 0.060; rank-biserial r = 0.263), with numerically smaller volumes in luminal lesions (median 0.760 vs. 5.990). Across diffusion histogram metrics, all ADC percentile measures and ADCmax were significantly lower in luminal lesions (ADCmin *p* = 0.031; ADC1–ADC99 all *p* < 0.001; ADCmax *p* = 0.003; rank-biserial r = 0.302–0.663), consistent with descriptives showing uniformly lower mean/median ADC values in the luminal group. Among shape descriptors, skewness differed significantly (*p* = 0.008; r = −0.371), indicating higher skewness in luminal lesions (median 0.661 vs. 0.144), whereas kurtosis (*p* = 0.352) and entropy (*p* = 0.493) did not differ significantly between groups.

Nonparametric group comparisons of ADC histogram metrics by biomarker status are presented in Supplementary Table S2.

### 3.3. Interobserver Agreement

To evaluate measurement reproducibility, a second radiologist independently repeated lesion segmentation and ADC histogram extraction. Interobserver agreement was good for the primary ADC predictors and lesion volume, with intraclass correlation coefficients (ICCs) of 0.852 for ADC10, 0.864 for ADC75, and 0.845 for lesion volume. These findings support the reproducibility of the ADC percentile metrics used in the prediction models.

### 3.4. Logistic Regression and ROC Performance

#### 3.4.1. ER Prediction

In univariable logistic regression (outcome: ER positivity; *n* = 72), lower ADC percentiles were consistently associated with higher odds of ER positivity, whereas histogram shape metrics were not. Among single-metric models, ADC10 provided the best fit (Akaike information criterion [AIC] 75.4; McFadden pseudo-R^2^ 0.278; *p* < 0.001).

In the multivariable model (Table 2) including age, log-transformed volume (log_volume), and ADC10×10, only ADC10×10 remained independently associated with ER positivity (OR 0.441; 95% confidence interval [CI] 0.289–0.672; *p* < 0.001), while age and log_volume were not significant. Model discrimination was good (AUC: 0.847). In ROC analysis based on the predicted ER probabilities (p_ER), AUC was 0.847 (95% CI 0.752–0.942); using the Youden index-optimized cutoff of p_ER = 0.671, the sensitivity was 0.725 (95% CI 0.587–0.863) and specificity was 0.906 (95% CI 0.805–1.000), yielding an accuracy of 0.806 and a balanced accuracy of 0.816 (Youden 0.631) (Table 3).

#### 3.4.2. PR Prediction

In univariable logistic regression (outcome: PR positivity; *n* = 72), lower ADC percentiles were consistently associated with higher odds of PR positivity, while histogram shape metrics were not clearly informative. Among single-metric models, ADC10 provided the best fit (AIC 78.4; McFadden pseudo-R^2^ 0.251; *p* < 0.001).

In the multivariable model (Table 2) including age, log-transformed volume (log_volume), and ADC10×10, only ADC10×10 remained independently associated with PR positivity (OR 0.478; 95% CI 0.321–0.713; *p* < 0.001), whereas age and log_volume were not significant; overall discrimination was good (AUC 0.819). In ROC analysis based on the predicted PR probabilities (p_PR), AUC was 0.819 (95% CI 0.720–0.918); using the Youden index-optimized cutoff of p_PR = 0.647, the sensitivity was 0.692 (95% CI 0.547–0.837) and specificity was 0.848 (95% CI 0.726–0.971), yielding an accuracy of 0.764 and a balanced accuracy of 0.770 (Youden 0.541) (Table 3).

#### 3.4.3. Ki-67 Prediction

In univariable logistic regression (outcome: Ki-67 positivity; *n* = 72), multiple ADC percentiles were significant predictors. Among single-metric models, ADC75 provided the best fit (AIC 41.8; McFadden R^2^ 0.302; *p* = 0.007).

In the multivariable model (Table 2) including age, log-transformed volume (log_volume), and ADC75×10, only ADC75×10 remained independently associated with Ki-67 positivity (OR 3.095; 95% CI 1.323–7.24; *p* = 0.009), while age and log_volume were not significant. Model performance was strong (AIC 41.2; McFadden R^2^ 0.387) with high discrimination (AUC 0.905). Using the Youden index-optimized cutoff of p_ki67 = 0.814, the sensitivity was 0.857 (95% CI 0.771–0.944) and specificity was 0.889 (95% CI 0.684–1.000), yielding an accuracy of 0.861 and a balanced accuracy of 0.873 (Youden 0.746) (Table 3).

#### 3.4.4. Luminality Prediction

In univariable logistic regression screening (outcome: non-luminal status; *n* = 72), multiple ADC histogram metrics—particularly ADC percentile features—were significant predictors, whereas kurtosis and entropy were not. Among single-metric models, ADC10 provided the best fit (AIC 75.8; McFadden R^2^ 0.260; *p* < 0.001) with good discrimination (AUC 0.829).

In the multivariable model (Table 2) including age, log-transformed volume (log_volume), and ADC10×10, only ADC10×10 remained independently associated with non-luminal status (OR 2.251; 95% CI 1.490–3.400; *p* < 0.001), while age and log_volume were not significant. Model performance remained good (AIC 78.6; McFadden R^2^ 0.273) with similar discrimination (AUC 0.832).

Using the Youden index-optimized cutoff on the model-predicted probability of non-luminal status (p_*n* = 0.382), the sensitivity was 0.828 (95% CI 0.690–0.965) and specificity was 0.814 (95% CI 0.698–0.930), yielding an accuracy of 0.819 and a balanced accuracy of 0.821 (Youden 0.642) (Table 3).

Binomial logistic regression models were fitted for ER, PR, Ki-67 and luminality status. Predictors included age, log-transformed lesion volume (log_volume), and the selected ADC percentile metric. ADC predictors were scaled by a factor of 10 (e.g., ADC10x10 = ADC10 × 10; ADC75x10 = ADC75 × 10) so that ORs correspond to a 0.1-unit increase in the original ADC scale. OR = odds ratio; CI = confidence interval; ADC = apparent diffusion coefficient.

## 4. Discussion

In this study, we evaluated whether DWI-derived ADC histogram metrics from breast cancer brain metastases were associated with primary tumor immunohistochemical biomarkers, focusing on estrogen receptor, progesterone receptor, human epidermal growth factor receptor 2, and the Ki-67 labeling index. Our main findings were as follows: (i) lower ADC values, particularly low-percentile metrics, were associated with ER and PR positivity; (ii) ADC histogram features did not show meaningful discrimination for HER2 status; and (iii) ADC metrics showed an exploratory signal for Ki-67 status in our dataset.

### 4.1. ER and PR Prediction: Emphasis on Low-Percentile Diffusion Metrics

Our finding that lower ADC metrics were associated with ER and PR positivity is directionally consistent with both the limited BCBM-specific literature and the broader primary breast cancer literature. In the key BCBM ADC histogram study, hormone receptor-positive metastases, reported as combined ER/PR positivity, showed significantly lower 25th-percentile ADC values. When all measurable metastases were included, additional histogram indices, including the 50th and 75th percentiles and mean ADC, were also lower in ER/PR-positive disease [8].

Although Ahn et al. [8] did not report diagnostic performance metrics for ER prediction in BCBM, the consistent direction of effect supports the biologic plausibility that lower diffusion may be associated with hormone receptor positivity in metastatic lesions [8]. More broadly, evidence from primary breast cancer supports the same pattern. Systematic reviews and meta-analyses have shown that ER-positive tumors tend to have lower ADC values than ER-negative tumors [4,5,12,13]. Similarly, PR-positive tumors have been reported to show lower ADC values than PR-negative tumors in primary breast cancer cohorts and meta-analytic syntheses [5,13,14,15,16].

The broad significance observed across correlated ADC percentiles in our dataset may reflect a global leftward shift of the ADC distribution rather than multiple independent signals. Taken together, our ER/PR findings are biologically plausible and align with the broader diffusion MRI literature, in which lower ADC, especially lower-end histogram percentiles, has been linked to microstructural features such as higher cellular density and reduced extracellular space in hormone receptor-positive disease [5,8,13].

### 4.2. HER2: Lack of Discriminatory Value for ADC Histograms in BCBM

In our analysis, ADC histogram metrics did not show a meaningful or consistent association with HER2 status. This finding is in line with the BCBM-specific literature, in which Ahn et al. [8] found no statistically significant differences across ADC histogram parameters between HER2-positive and HER2-negative brain metastases, despite clear ER/PR-related differences within the same cohort [8]. In our cohort, no ADC percentile metric differed significantly by HER2 status, and effect sizes were uniformly small across the evaluated ADC metrics. Together, these findings support the absence of a meaningful ADC histogram signal for HER2 in this setting. Biologically, this lack of association may reflect the combined influence of HER2-related vascularity and permeability, cellularity-related diffusion restriction, and metastatic microenvironmental factors such as edema, necrosis, and treatment-related change, all of which may blur diffusion-based contrasts in brain metastases [8].

Although some studies in primary breast cancer have reported modest ADC differences according to HER2 status, these associations have been inconsistent and may not extrapolate directly to brain metastases [4,5,12,17,18]. Taken together, the concordance between our findings and the limited BCBM-focused literature suggests that conventional ADC histogram features, as currently implemented, are unlikely to serve as reliable stand-alone imaging markers of HER2 status in metastatic brain disease.

### 4.3. Ki-67: Biological Interpretation and the Role of Percentile Selection

We observed a Ki-67-related signal in our dataset; however, this finding should be interpreted cautiously. Direct evidence in breast cancer brain metastases remains limited because the only dedicated BCBM ADC histogram study evaluated ER/PR and HER2, but not Ki-67 [8]. Accordingly, biologic interpretation of our Ki-67 result relies mainly on related evidence from mixed-primary brain metastasis cohorts, lung cancer brain metastases, and primary breast tumors. In those settings, ADC metrics generally show an inverse relationship with Ki-67, with low- and mid-range ADC percentiles typically providing stronger proliferation-related information than upper-percentile metrics [19,20,21,22]. This differs from our finding that ADC75 provided the strongest signal and suggests that our result should be regarded as preliminary rather than definitive.

A plausible explanation is both methodological and biological. In our study, a whole-lesion segmentation strategy was used, and cystic, necrotic, and hemorrhagic intralesional components were not intentionally excluded. Although this approach preserves intralesional heterogeneity, it may also increase upper-percentile ADC values by incorporating non-cellular components with relatively facilitated diffusion. As a result, higher-percentile metrics such as ADC75 may reflect mixed contributions from viable tumor, necrosis, cystic change, hemorrhagic products, and other microenvironmental features rather than cellular proliferation alone [8,19,20]. This may partly explain why the Ki-67-related signal in our cohort emerged at a higher percentile than would be expected from the broader diffusion–proliferation literature. In addition, the relatively small number of Ki-67-negative cases may have increased instability in the estimated diagnostic performance. Taken together, these Ki-67 findings should be considered exploratory and hypothesis-generating and require confirmation in larger cohorts with independent validation and, ideally, lesion-level metastatic pathology.

### 4.4. Luminality: ADC10-Based Discrimination of Luminal vs. Non-Luminal Breast Cancer Brain Metastases

In this cohort, ADC10 was associated with luminal status. In multivariable binomial logistic regression adjusted for age and log-transformed lesion volume, ADC10×10 remained independently associated with non-luminal status. Discrimination was moderate to good within this cohort (AUC = 0.832), and the Youden index-optimized probability threshold yielded relatively balanced sensitivity and specificity. However, this result should be interpreted as an internally derived estimate from an exploratory single-cohort analysis rather than as a validated subtype-classification tool.

These findings are directionally consistent with the limited brain metastasis-specific ADC histogram literature. Ahn et al. [8] reported that the 25th percentile was significantly lower in estrogen receptor- and/or progesterone receptor-positive disease than in ER/PR-negative disease, and that additional central tendency measures also separated ER/PR groups when all measurable metastases were segmented, whereas human epidermal growth factor receptor 2 showed no significant differences [8]. Our ADC10-based model is broadly concordant with that observation in suggesting that lower-tail diffusion information may carry a biologically relevant signal. However, the present findings should be viewed as complementary rather than directly equivalent, given differences in percentile selection, modeling strategy, and outcome definition.

When interpreted in the broader literature from primary breast tumors, the present results are also compatible with the general concept that ADC-derived measures may correlate with receptor-defined phenotypes, although effect sizes and individual-level separability vary across cohorts and measurement strategies. For example, Horvat et al. showed that ADC metrics differed by ER/PR status and reported only moderate discrimination for luminal versus non-luminal classification (AUC ≈ 0.685) using maximum whole-tumor ADC, highlighting substantial overlap between subtypes at the individual level [4]. More pessimistic syntheses also exist. A systematic review and meta-analysis concluded that mean ADC values overlap substantially across molecular subtypes and are not sufficient for reliable subtype discrimination [6]. A large multicenter analysis similarly found that ADC could not predict molecular subtype or nodal status in invasive breast cancer [7]. Against that background, the comparatively higher AUC observed here may reflect the use of a low-percentile feature (ADC10), biological and microenvironmental differences in brain metastases, and/or optimistic performance related to single-cohort threshold optimization, underscoring the need for external validation.

The biological plausibility of low-percentile ADC as a discriminative marker is also supported by work in other brain metastasis contexts. In lung cancer brain metastases, histogram-derived low percentiles, including ADC10 and ADC25, have achieved high AUCs for distinguishing histologies such as small-cell versus non-small-cell disease, supporting the view that lower-tail diffusion may better capture the most cellular or diffusion-restricted compartments [19,20]. Methodologically, the minimal gain observed after adding covariates in our study (AUC 0.829 in the univariable model vs. 0.832 in the multivariable model) is also in line with prior reports suggesting that basic clinical or imaging covariates often provide only incremental, rather than dramatic, improvements in subtype-related models [23].

### 4.5. Clinical Relevance: Toward Non-Invasive Biomarker Estimation in BCBM

From a clinical perspective, non-invasive assessment of tumor biology in BCBM is of interest because receptor profiles may be discordant or may change between primary tumors and metastases, and repeated tissue sampling can be challenging. MRI-based approaches for receptor tracking and radiomic prediction have been proposed specifically for BCBM [2,3]. In this context, our findings suggest that diffusion-derived ADC histogram features, particularly low-percentile ADC metrics, may provide candidate imaging correlates of ER/PR-related biology, whereas HER2 likely requires richer multiparametric approaches beyond conventional ADC histograms alone [3,8]. However, these observations should be interpreted in light of the fact that our reference standard was derived from the primary tumor rather than the brain metastasis itself.

### 4.6. Thresholds, AUC, and Pperformance Reporting

Finally, our results should be interpreted in light of modern guidance on model evaluation under imbalance. ROC-derived area under the curve (AUC) reflects ranking ability and is relatively insensitive to prevalence; consequently, apparently high AUC values may coexist with clinically limited threshold-dependent performance for the minority class [24,25,26,27]. Prior work has emphasized that reliance on a default probability cutoff may bias predictions toward the majority class in imbalanced medical imaging settings, and that explicit threshold optimization may be needed to better align model outputs with clinical priorities [28,29,30,31]. In the present study, both model development and Youden-based threshold selection were performed within the same cohort. Therefore, these performance estimates should be regarded as internally derived and potentially optimistic rather than ready for clinical use.

### 4.7. Limitations

This study has several limitations. First, the retrospective single-center design and single-cohort analysis limit generalizability. Second, the reference standard was derived from the primary breast tumor rather than the brain metastasis itself; therefore, our findings reflect associations between metastatic ADC histogram features and primary tumor biomarker status rather than direct imaging-based characterization of metastatic receptor status. Third, only one lesion per patient was analyzed, and the largest metastasis was selected as a pragmatic strategy to avoid within-patient clustering. However, this approach may not fully capture intrapatient heterogeneity in multifocal brain metastatic disease [8]. Fourth, lesions that were not reliably evaluable on DWI/ADC maps were excluded, which may have favored better-delineated lesions and introduced selection bias. Fifth, segmentation was performed directly on ADC maps to avoid registration-related error. However, lesion conspicuity is generally better on contrast-enhanced T1-weighted images, and direct ADC-based segmentation may reduce boundary clarity and comparability with CE-T1-defined workflows. Sixth, our whole-lesion VOIs intentionally included cystic, necrotic, and hemorrhagic components to preserve intralesional heterogeneity. Although this strategy reflects biologic complexity, it may have influenced absolute histogram values, particularly upper ADC percentiles, and may limit interpretation of these metrics as surrogates of viable tumor cellularity alone [8,19,20]. Seventh, Ki-67-related interpretation remains indirect in BCBM because dedicated breast cancer brain metastasis ADC histogram data are sparse, and prior diffusion–proliferation evidence comes mainly from mixed-primary brain metastases, lung cancer brain metastases, or primary breast tumors. Moreover, the relatively small number of Ki-67-negative cases may have reduced the stability of the performance estimates [8,19,20]. Eighth, treatment information was not comprehensively available, and prior local or systemic therapies may have altered diffusion characteristics. Ninth, luminality was operationalized as ER and/or PR positivity, which is a simplified surrogate rather than a full contemporary intrinsic subtype classification. Tenth, reproducibility was assessed only for the primary predictors retained in the final models and lesion volume, not for the full set of screened histogram features. Finally, the analysis was exploratory and involved multiple comparisons and feature selection within the same dataset. Accordingly, the reported AUCs and Youden-derived thresholds may be optimistic and should not be interpreted as validated clinical cutoffs without independent external validation and calibration assessment [27,29].

## 5. Conclusions

ADC histogram analysis derived from DWI showed exploratory associations with hormone receptor status in breast cancer brain metastases, with low-percentile diffusion metrics providing the strongest signal for ER and PR characterization. In contrast, conventional ADC histogram features did not show a meaningful or consistent association with HER2 status in this cohort. Ki-67-related findings should be interpreted cautiously because of the limited disease-specific evidence, the inclusion of heterogeneous whole-lesion components, and the potential instability introduced by class imbalance. Overall, these findings suggest that ADC histogram features may serve as candidate imaging biomarkers of primary tumor biology in breast cancer brain metastases. However, they should not be interpreted as a substitute for metastatic tissue characterization. Rather, they should be viewed as indirect imaging correlates that may inform hypothesis generation and future multimodal biomarker research. Further confirmation in larger multicenter cohorts is needed, with prespecified endpoints, appropriate control for multiple testing, external validation, and calibration assessment before any clinical application can be considered.

## Figures and Tables

**Table 1:** Baseline characteristics of the cohort and lesions.

Characteristic	Overall (*n* = 72)
Patient characteristics	
Age, years	54.0 (15.25) [33–77]
Lesion imaging characteristics	
Maximum diameter, mm	18.3 (19.05) [3.2–83.7]
Lesion volume	1.102 (6.599) [0.046–114.09]
ADC min	0.593 (0.194) [0.381–1.060]
ADC 1st percentile	0.681 (0.219) [0.400–1.120]
ADC 5th percentile	0.747 (0.267) [0.400–1.210]
ADC 10th percentile	0.800 (0.262) [0.463–1.360]
ADC 25th percentile	0.885 (0.370) [0.500–1.660]
ADC 50th percentile	1.006 (0.468) [0.500–2.550]
ADC 75th percentile	1.113 (0.657) [0.600–2.900]
ADC 90th percentile	1.298 (0.887) [0.600–2.990]
ADC 95th percentile	1.418 (1.007) [0.672–3.100]
ADC 99th percentile	1.633 (1.076) [0.672–3.330]
ADC max	1.637 (1.130) [0.672–4.090]
Skewness	0.426 (0.739) [−0.583–2.010]
Kurtosis	−0.522 (1.300) [−1.514–4.870]
Entropy	3.859 (0.539) [2.138–4.420]
Biopsy/pathology characteristics	
Biopsy type	
—Excisional	36 (50.0%)
—Incisional	36 (50.0%)
Histopathologic subtype	
—IDC (invasive ductal carcinoma)	66 (91.7%)
—ILC (invasive lobular carcinoma)	3 (4.2%)
—Mixed	3 (4.2%)
Histologic grade	
—Grade 1	2 (2.8%)
—Grade 2	20 (27.8%)
—Grade 3	50 (69.4%)
Estrogen receptor (ER)	
—Negative	32 (44.4%)
—Positive	40 (55.6%)
Progesterone receptor (PR)	
—Negative	33 (45.8%)
—Positive	39 (54.2%)
Human epidermal growth factor receptor 2 (HER2)	
—Negative	40 (55.6%)
—Positive	32 (44.4%)
Molecular subtype	
—HER2-enriched	13 (18.1%)
—Luminal B—HER2-negative	23 (31.9%)
—Luminal B—HER2-positive	20 (27.8%)
—Triple-negative	16 (22.2%)
Ki-67	
—Negative	9 (12.5%)
—Positive	63 (87.5%)
Lymphovascular invasion (LVI)	
—Negative	11 (15.3%)
—Positive	61 (84.7%)

Continuous variables are presented as median (IQR) [min–max]; categorical variables as n (%). ADC values are expressed in ×10^−3^ mm^2^/s, and lesion volume is expressed in cm^3^ (mL). ER = estrogen receptor; PR = progesterone receptor; HER2 = human epidermal growth factor receptor 2; ADC = apparent diffusion coefficient; LVI = lymphovascular invasion; IDC = invasive ductal carcinoma; ILC = invasive lobular carcinoma.

**Table 2:** Multivariable logistic regression models for biomarker and luminality prediction using ADC histogram percentiles.

ER Model (ER = P vs. N): Age + log_volume + ADC10x10			
Predictor	OR	95% CI	*p*-value
Age	0.989	0.935–1.046	0.697
log_volume	1.137	0.790–1.637	0.489
ADC10x10	0.441	0.289–0.672	<0.001
PR model (PR = P vs. N): age + log_volume + ADC10x10			
Age	0.991	0.938–1.046	0.734
log_volume	1.086	0.761–1.549	0.649
ADC10x10	0.478	0.321–0.713	<0.001
Ki-67 model (Ki-67 = P vs. N): age + log_volume + ADC75x10			
Age	0.928	0.831–1.040	0.182
log_volume	0.590	0.246–1.410	0.237
ADC75x10	3.095	1.323–7.240	0.009
Luminality model (N vs. L): age + log_volume + ADC10x10			
Age	1.007	0.952–1.065	0.811
log_volume	0.816	0.564–1.181	0.281
ADC10x10	2.251	1.490–3.400	<0.001

**Table 3:** Discrimination performance and Youden-optimized probability thresholds.

Outcome	Predictor (Model Probability)	AUC (95% CI)	Youden Optimal Cutoff	Sensitivity	Specificity	Accuracy
ER (P vs. N)	p_ER	0.847 (0.752–0.942)	0.671	0.725	0.906	0.806
PR (P vs. N)	p_PR	0.819 (0.720–0.918)	0.647	0.692	0.848	0.764
Ki-67 (P vs. N)	p_ki67	0.905 (0.820–0.989)	0.814	0.857	0.889	0.861
Luminality (N vs. L)	p_N	0.832 (0.730–0.935)	0.382	0.828	0.814	0.819

AUC = area under the receiver operating characteristic curve; CI = confidence interval; ER = estrogen receptor; PR = progesterone receptor; p_ER/p_PR/p_ki67/p_N denote model-predicted probabilities. Youden cutoffs were derived on the study cohort to maximize (Sensitivity + Specificity − 1). AUC confidence intervals were estimated via bootstrap resampling in the ROC module settings.

## Data Availability

The data presented in this study are available from the corresponding author upon reasonable request, subject to institutional review and applicable ethical and privacy restrictions.

## Supplementary Materials

The following supporting information can be downloaded at https://www.mdpi.com/article/10.3390/diagnostics16081154/s1, Table S1: Standardized institutional brain metastasis MRI protocol; Table S2: Mann–Whitney U test *p*-values for ADC histogram metrics according to biomarker and luminality status, with additional rank-biserial effect sizes for HER2 comparisons.

## Abbreviations

The following abbreviations are used in this manuscript:

ADC	apparent diffusion coefficient
AIC	Akaike information criterion
ASCO/CAP	American Society of Clinical Oncology/College of American Pathologists
AUC	area under the receiver operating characteristic curve
BCBM	breast cancer brain metastases
CI	confidence interval
DWI	diffusion-weighted imaging
ER	estrogen receptor
FLAIR	fluid-attenuated inversion recovery
HER2	human epidermal growth factor receptor 2
ICC	intraclass correlation coefficient
IQR	interquartile range
Ki-67	Ki-67 proliferation index
log_volume	log-transformed lesion volume
MRI	magnetic resonance imaging
N	negative (biomarker status)
OR	odds ratio
P	positive (biomarker status)
p_ER	model-predicted probability of ER positivity
p_ki67	model-predicted probability of Ki-67 positivity
p_N	model-predicted probability of non-luminal status
p_PR	model-predicted probability of PR positivity
PR	progesterone receptor
ROC	receiver operating characteristic
ROI	region of interest
T1WI	T1-weighted imaging
T2WI	T2-weighted imaging
VOI	volume of interest
