# Supplementary material for: ADC Histogram Features of Breast Cancer Brain Metastases as Candidate Imaging Biomarkers of Primary Tumor ER, PR, Ki-67, and Luminal Status
Source: Diagnostics (Basel). 2026 Apr 13;16(8):1154. doi: 10.3390/diagnostics16081154 (PMC13114955; doi:10.3390/diagnostics16081154)
Supplement: Supplementary file 1 [file diagnostics-16-01154-s001.zip › diagnostics-4208891-supplementary.pdf]

Supplementary Table S1. MRI acquisition protocol parameters

	TE/TR (ms)	Slice Thickness(mm)	FOV(cm)	Matrix
Axial FLAIR FS	104.46/9000	4	22x22	288x192
Axial T1 W	8.76/509	4	22x22	224x224
Axial T2 W	127.56/4082	4	22x22	416x416
Sagittal T2 W	114.6/5638	5	24x24	352x352
Coronal T2* GRE	18/660	5	23x23	320x192
CE 3D Sagittal T1 CUBE	13.94/502	1.2	25.6x25.6	320x192
CE Axial T1 W	8.76x509	4	22x22	224x224
Axial DWI (b=0/1000)	93.2/7702	4	22x22	160x160

MRI: magnetic resonance imaging; TE: echo time; TR: repetition time; FOV: field of view; FS: fat-saturated; T1 W: T1-weighted; T2 W: T2-weighted; GRE: gradient echo; CE: contrast-enhanced; DWI: diffusion-weighted imaging.

Supplementary Table S2. Mann-Whitney U test p-values for ADC histogram metrics according to biomarker and luminality status, with additional rank-biserial effect sizes for HER2 comparisons.

**Metric**	**ER status (P vs N)**	**PR status (P vs N)**	**HER2 status (P vs N)**	**HER2 rank-biserial r**	**Ki-67 status (P vs N)**	**Luminality statu (luminal vs non-**
ADCmin	0.024	0.021	0.964	-0.007	0.045	0.031
ADC1	<0.001	<0.001	0.515	0.091	0.003	<0.001
ADC5	<0.001	<0.001	0.489	0.096	<0.001	<0.001
ADC10	<0.001	<0.001	0.448	0.105	<0.001	<0.001
ADC25	<0.001	<0.001	0.415	0.113	<0.001	<0.001
ADC50	<0.001	<0.001	0.318	0.139	<0.001	<0.001
ADC75	<0.001	<0.001	0.386	0.120	<0.001	<0.001
ADC90	<0.001	<0.001	0.511	0.091	<0.001	<0.001
ADC95	<0.001	<0.001	0.395	0.118	<0.001	<0.001
ADC99	<0.001	<0.001	0.401	0.117	<0.001	<0.001
ADCmax	0.001	<0.001	0.356	0.128	<0.001	0.003
Skewness	0.032	0.047	0.579	-0.077	0.658	0.008
Kurtosis	0.650	0.422	0.812	0.034	0.695	0.352
Entropy	0.242	0.114	0.757	0.044	0.563	0.493

Mann-Whitney U test p-values are reported for all comparisons. For HER2 comparisons, rank-biserial correlation coefficients (r) are additionally provided to indicate effect size.
Abbreviations: ADC, apparent diffusion coefficient; ER, estrogen receptor; PR, progesterone receptor; HER2, human epidermal growth factor receptor 2.
